# Supplementary material for: Unraveling dynamic factors in Dialectic Behavioural Treatment for Adolescents (DBT-A): A study protocol
Source: PLoS One. 2026 Jul 16;21(7):e0340173. doi: 10.1371/journal.pone.0340173 (PMC13375012; doi:10.1371/journal.pone.0340173)
Supplement: S1 Appendix — (DOCX) [file pone.0340173.s001.docx]

**S1. Reference list topic list (pilotstudy)**

This Supplementary material belongs to:

**Article title:** Unraveling dynamic factors in Dialectic Behavioural Treatment for Adolescents (DBT-A): a study protocol.

**Journal name:** PLOS ONE

**Author names**: Anneke de Weerd, Anne A. Krabbendam, Agaath Koudstaal, Joep Sins, Jacquelijne Schraven, Robert R.J.M. Vermeiren, Jantine Roeleveld, Elisabeth Koopman-Verhoeff, Laura A. Nooteboom

**Affiliation of corresponding author**: Anneke de Weerd, LUMC Curium–Department of Child and Adolescent Psychiatry, Leiden University Medical Center, Post Box 15, 2300 AA, Leiden, The Netherlands

**E-mail address of corresponding author**: a.de_weerd@lumc.nl

This supplementary information presents the literature reviewed to develop the topic list, clinical perspective, and experiential perspective on the subject.

S1. Topic list: Integration of scientific, clinical and experiential knowledge

| **Topic** | **Description from Literature (References)** | **Clinical Perspective** | **Experiential Perspective** |
| --- | --- | --- | --- |
| Therapeutic Alliance: General | Description of the relationship between the therapist and the youth/parent [1-3]. | Alliance with different therapists (individual and systemic); level of support; dependency on the therapist; fluctuations in closeness/distance. | Interpretation by the youth; recognition and being heard by youth and parents; transparency of intent; authenticity (feeling and showing); balance between validation and boundaries. |
| Responsiveness | How the therapist can time, use intuition, validate, and show empathy in interactions [4, 5]. | Irreverence (validation vs. confrontation); levels of validation; tuning in; labeling; containing. | Recognition of the youth’s interpretation; authenticity and clarity; balance between validation and setting limits. |
| Attitude of Those Involved | Attitudes (and behaviors) of those involved, and their influence on relationships/dynamics [5]. | Fluctuating therapist attitudes; dialectical dilemmas; humor; validating environment; curiosity about others. | Roles and functions of all involved; explanation of impact of attitudes; inter-therapist attitudes; group climate also influential. |
| Feelings Toward One Another | Emotions towards all involved and their influence on relationships/dynamics [6, 7]. | Expressing negative emotions (as therapist); transference/countertransference; working through emotions; tolerating vs. internalizing emotions. | Shifting feelings; constructively sharing emotions; recognizing emotions. |
| (Response to) Specific Characteristics of the Target Group | Reactions to traits of youth with BPD, such as lack of trust, idealization/devaluation, impulsivity [8]. | Splitting behavior; distrust/disconnection; feelings of shame and rejection; de-shaming. | Internalization of others’ reactions; stigmatization; diagnosis is not an excuse; role of adolescence; treatment goals as pressure; agreement on treatment goals. |
| Processing and Discussing Relationship Ruptures | E.g., withdrawal or confrontation and mutual reactions [9]. | Ruptures initiated by therapist; youth/parent’s view on rupture; focus on relationship repair; personalizing detachment behavior. | Adolescence phase; importance of humor; constructively discussing temporary ruptures. |
| Treatment Expectations and Motivation | Motivation and confidence in treatment from all involved [10, 11]. | Clarity on treatment course, goals, and expected outcomes; treatment perceived as meaningful; parents’ role in motivation. | Fluctuating motivation; role of parents, social network, group climate; repeating expectations; treatment history; misinterpretation of demotivation; shared vision of success. |
| Communication Style of Therapist | Active, passive; adaptability to youth and parent behavior [1, 12]. | Flexibility (switching between validating and confronting). | Importance of “click,” openness about style, flexibility. |
| Sense of Connection / (Dis)connection with Others | Youth’s sense of connection to others [8, 13, 14]. | Includes therapist, family, social network; identification with therapist; feelings of loneliness. | Make layers of connection concrete; group climate; space for connection. |
| Sense of Connection / (Dis)connection with Self | Youth’s self-connection [8, 15]. | Identity formation/adolescence phase; learning to connect with self in small steps; therapist’s labeling. | Prerequisite for treatment progress. |
| Sense of Skill Improvement | E.g., emotion regulation and self-control [2]. | Reinforcing environment; different views on improvement. | Self-confidence; unconscious progress; small successes; celebrating wins; applying in daily life; what if it goes worse?; different views on progress. |
| Therapist Self-disclosure | Degree to which therapist shares personal experiences [16]. | Effect on youth and parents (more openness/less shame?); impact on relationship (more honesty?). | Timing; concrete examples; role of peer workers. |
| Role/Function of Parents in Treatment | – | Sense of control and involvement for parents; respecting their position as caregivers. | Role of parents in treatment; working on parents’ own goals. |
| Load Capacity and Resilience | – | Load capacity affects treatment. | Capacity of parents/youth affects autonomy and treatment success. |
| Collaboration with Other Practitioners | – | – | Coordination across all practitioners, including outside DBT team. |
| Reflective Capacity | – | – | Youth and therapist must both reflect on treatment and relationship. |
| Crisis Behavior / Hopelessness | – | Changes in crisis behavior/hopelessness and their effect on those involved (therapist, parents, youth). | – |
| Overall Functioning / Stability | – | Level of stability and living situation of youth (e.g., housing). | Consider overall mental state/life events/environmental stability. |

**References (topic list)**

1 De Soet R, Vermeiren RRJM, Bansema CH, Van Ewijk H, Nijland L, Nooteboom LA (2023) Drop-out and ineffective treatment in youth with severe and enduring mental health problems: a systematic review. Eur Child Adolesc Psychiatry 33(10):3305-3319. https://doi.org/10.1007/s00787-023-02182-z

2 Rudge S, Feigenbaum JD, Fonagy P (2017) Mechanisms of change in dialectical behaviour therapy and cognitive behaviour therapy for borderline personality disorder: a critical review of the literature. J Ment Health 29(1):92-102. https://doi.org/10.1080/09638237.2017.1322185

3 Horvath AO, Del Re AC, Flückiger C, Symonds D (2011) Alliance in individual psychotherapy. Psychother (Chic) 48(1):9-16. https://doi.org/10.1037/a0022186

4 Hatcher RL (2015) Interpersonal competencies: Responsiveness, technique, and training in psychotherapy. Am Psychol 70(8):747-757. https://doi.org/10.1037/a0039803

5 Hutsebaut J, Bachrach N, Kindt KCM, van Dam LJH (2022) Hoe bewezen effectief is de guideline-informed treatment for personality disorders (GIT-PD)? Tijdschr Psychiatr 64(1):18-24.

6 Hoffart A, Friis S (2000) Therapists’ emotional reactions to anxious inpatients during integrated behavioral-psychodynamic treatment: a psychometric evaluation of a feeling word checklist. Psychother Res 10(4):462-473. https://doi.org/10.1093/ptr/10.4.462

7 Holmqvist R (2000) Associations between staff feelings toward patients and treatment outcome at psychiatric treatment homes. J Nerv Ment Dis 188(6):366-371. https://doi.org/10.1097/00005053-200006000-00007

8 Morris C, Smith I, Alwin N (2014) Is contact with adult mental health services helpful for individuals with a diagnosable BPD? A study of service users views in the UK. J Ment Health 23(5):251-255. https://doi.org/10.3109/09638237.2014.951483

9 Zimmermann R, Krause M, Weise S, Schenk N, Fürer L, Schrobildgen C, Schlüter-Müller S, Valdes N, Koenig J, Kaess M, Schmeck K (2018) A design for process-outcome psychotherapy research in adolescents with borderline personality pathology. Contemp Clin Trials Commun 12:182-191. https://doi.org/10.1016/j.conctc.2018.10.007

10 Greenberg RP, Constantino MJ, Bruce N (2005) Are patient expectations still relevant for psychotherapy process and outcome? Clin Psychol Rev 26(6):657-678. https://doi.org/10.1016/j.cpr.2005.03.002

11 Bäumer A, Fürer L, Birkenberger C, Wyssen A, Steppan M, Zimmermann R, Gaab J, Kaess M, Schmeck K (2022) The impact of outcome expectancy on therapy outcome in adolescents with borderline personality disorder. Borderline Personal Disord Emot Dysregul 9(1). https://doi.org/10.1186/s40479-022-00200-1

12 Ryan R, Berry K, Hartley S (2021) Therapist factors and their impact on therapeutic alliance and outcomes in child and adolescent mental health – a systematic review. Child Adolesc Ment Health 28(2):195-211. https://doi.org/10.1111/camh.12518

13 Barzilay S, Feldman D, Snir A, Apter A, Carli V, Hoven C, Wasserman C, Sarchiapone M, Wasserman D (2015) The interpersonal theory of suicide and adolescent suicidal behavior. J Affect Disord 183:68-74. https://doi.org/10.1016/j.jad.2015.04.047

14 De Bruin R, Koudstaal A, Muller N (2013) Surfen op Emoties: werkboek dialectische gedragstherapie voor jongeren (DGT-J). Houten: Bohn Stafleu van Loghum: Kind en Adolescent Praktijk.

15 Bungert M, Liebke L, Thome J, Haeussler K, Bohus M, Lis S (2015) Rejection sensitivity and symptom severity in patients with borderline personality disorder: effects of childhood maltreatment and self-esteem. Borderline Personal Disord Emot Dysregul 2(1). https://doi.org/10.1186/s40479-015-0025-x

16 Weinberg I, Ronningstam E, Goldblatt MJ, Schechter M, Maltsberger JT (2010) Common factors in empirically supported treatments of borderline personality disorder. Curr Psychiatry Rep 13(1):60-68. https://doi.org/10.1007/s11920-010-0167-x
