# Supplementary material for: Unraveling dynamic factors in Dialectic Behavioural Treatment for Adolescents (DBT-A): A study protocol
Source: PLoS One. 2026 Jul 16;21(7):e0340173. doi: 10.1371/journal.pone.0340173 (PMC13375012; doi:10.1371/journal.pone.0340173)
Supplement: Attachment — Submitted filename: 3.1.3.4.2 Response letter_V1.pdf [file pone.0340173.s003.pdf]

15-12-2025

Dear Editor, Dear Joven Serdoncillo,

Thank you for considering the manuscript. An English translation of the entire original ethics approval document is uploaded as a Supporting Information file.

Kind regards,

On behalf of all authors,

Anneke de Weerd
