# Supplementary material for: Unraveling dynamic factors in Dialectic Behavioural Treatment for Adolescents (DBT-A): A study protocol
Source: PLoS One. 2026 Jul 16;21(7):e0340173. doi: 10.1371/journal.pone.0340173 (PMC13375012; doi:10.1371/journal.pone.0340173)
Supplement: Attachment — Submitted filename: Response to Reviewers.pdf [file pone.0340173.s004.pdf]

Academic Editor Dr. Na

Plos One

Responsletter on manuscript [PONE-D-25-63596]

March 29, 2026

Dear Dr. Na,

Thank you for giving us the opportunity to submit a revised version of our study protocol titled "Unraveling dynamic factors in Dialectic Behavioural Treatment for Adolescents (DBT-A): a study protocol" (PONE-D-25-63596). We sincerely appreciate the time and effort you and the reviewers have dedicated to reviewing our work. The constructive feedback has been highly valuable and has helped us to significantly improve the quality and clarity of our protocol.

We have carefully considered all the comments provided by the reviewers and have made the necessary improvements and additions to the manuscript in the point-by-point responses below.

We have also uploaded both a clean copy of the revised manuscript and a version with 'tracked changes' so that the modifications are easy to identify. Additionally, we have included the interview guide as a supplementary file. This document outlines the specific questions that will be asked to the participants, systematically organized by topic.

We hope that these revisions adequately address the reviewers' concerns and that the manuscript is now suitable for publication in Plos One.

Thank you again for your time, guidance, and consideration. We look forward to hearing from you.

Kind regards,

On behalf of all authors,

Anneke de Weerd

**Reviewer #1**: Dear Authors,
Thank you for the opportunity to review your study protocol. I would like to begin by acknowledging the significant value of this work.
Below are my formal comments and the issues that should be addressed before the protocol can be considered for publication.

Dear Reviewer 1,

Thank you for your thorough review and your highly insightful comments. Your feedback has been invaluable in adding necessary depth to this section of our protocol. See below our response/adaptions to the manuscript.

1. Rationale and Research Questions
The manuscript provides a valid and well-justified rationale. By moving beyond aggregate quantitative data to explore individual "trajectories," you address a critical gap in understanding why some adolescents do not respond to DBT-A. The research questions are clearly stated and align with the proposed qualitative longitudinal design.

Thank you.

2. Methodology and Replicability
The protocol is technically sound and the use of Reflexive Thematic Analysis (RTA) combined with Framework Analysis is appropriate. However, to ensure full replicability, the following points must be addressed:

1. Interview Guides: While you identify themes like "alliance" and "motivation," the specific semi-structured interview prompts are not included. Please provide the interview topic guides as supporting information.

We added an additional file with the interview prompts included for all participants groups (see supporting information 2). We added a reference in the study protocol to the document in Method-materials at line 356.

2. Definition of a "Case": Given the high-risk nature of this population, dropout is likely. You must explicitly define what constitutes a "complete case" for your analysis. If a parent or therapist leaves the study but the adolescent remains, will that trajectory still be included? Clarifying the "minimum data set" is essential to prevent

undisclosed flexibility in the analysis pipeline.

We added the sentence: *A complete case is defined by a minimum of three measurements moments per participant group (e.g. adolescent, parent and individual therapist). Although incomplete cases/trajectories are excluded from the longitudinal analysis, their individual data points will be included in thematic sub-analyses to maximize data utility. (*see Participants lines 284-287).

3. Feasibility and Potential Biases

*3.* Recruitment Bias: Because therapists recruit their own patients, there is a significant risk of "cherry-picking" stable or "likable" cases. I suggest the authors describe a more randomized recruitment approach (e.g., inviting the next three eligible patients) to ensure the data includes the "difficult" dynamics the study aims to unravel.

Based on the eligibility criteria, these contacts will screen all adolescents admitted to DBT-A treatment between 2025 and 2026 and all will be invited to participate in order to minimize selection bias. We also added: *The contact person at each participating institution will maintain a screening record of all potential participants, documenting their eligibility status and the rationale for inclusion or exclusion (lines 299-302)*

4. Parental Dyads: You mention a preference for interviewing parents together. This "dyadic" approach can suppress conflicting views or domestic tensions. Please justify this choice or mention a protocol for offering individual "break-out" sessions if tension is observed.

We rewrote and added the advise to: *Parents are offered the choice of joint or individual participation. Although both parents will be interviewed together in a dyadic format where possible, individual interviews will be arranged if a joint session is not preferred. If participation from both parents is not feasible, the involvement of a single parent is sufficient*. (see method-participants lines 261-265).

4. Data Availability and Ethics

5. PLOS Data Policy: You have correctly identified that the data is sensitive and will require restricted access. However, for publication, you must provide contact information for an independent Data Access Committee or Ethics Committee. Relying solely on "available upon request from the authors" does not meet current

transparency standards.

We added the sentence: *The pseudonymized datasets generated during the current study will be available from the LUMC Curium data officer upon reasonable request, subject to consultation with the authors.* (meta data-see lines 27-29).

6. Sentence Completion: Please review page 13; the sentence ending in "...and to" appears to be cut off.

We have re-read the manuscript but cannot find the sentence completion issue the reviewer is pointing at.

5. Language and Presentation
The manuscript is written in high-quality standard English. Your disclosure regarding the use of AI for translation and linguistic refinement is appreciated and aligns with ethical transparency, provided you maintain full accountability for the technical content.

Thank you, we indeed take full accountability for the technical content, and we have added this to the statement (metadata- see line 35).

7. Optional Suggestions for the Study Plan
   Member Checking: To mitigate attrition over the 9-month period, consider offering participants a brief, clinically appropriate summary of the general themes found in the study as an incentive for their continued contribution.

Thank you for this suggestion. To further explain the procedure, the expert by experience and the project team, representing all four participating institutions, will validate the themes emerging from the analysis. Participants will be offered the opportunity to review their transcripts for accuracy and provide clarifications upon completion of the data collection. Furthermore, the overall study results will be shared with participants. Given the longitudinal design and the need to avoid biasing participants during the nine-month study period, this information will be provided only after the study has concluded. This has been noted in lines the protocol, under the 'Procedure' section lines 326-328.

8. Power Dynamics: In the triadic model, the researcher must be careful not to be perceived as "part of the clinical team." Clearly emphasizing the independence of the research team during the consent process will be vital for obtaining honest critiques of the therapy.

We added the following information to emphasize the independence: *The researcher will explicitly declare her independence from the clinical team to mitigate power imbalances. Additionally, participants will be assured of intra-triadic confidentiality, clarifying that no*

*information provided by one participant will be disclosed to the others during the research process.* (see lines 310-313 in procedure).

**Reviewer #2:** A very interesting study with relevant objectives. Congratulations!
Dear Reviewer 2,

Thank you for the points raised below. Your detailed feedback offered a valuable perspective, allowing us to strengthen and elaborate on several aspects of our methodology.

Methodology:

9. It would be important to better describe the type of study. From what I understand, it is a mixed, prospective study with interviews, questionnaires, and scales.

We added: *This study adopts a prospective, longitudinal multiple-case design with an embedded mixed methods approach. A qualitatively-driven interpretation will be employed, placing primary emphasis on the qualitative findings, while quantitative data serve a supportive, complementary role.* Lines 201-204

10. Why not put it in a flowchart? To better understand the study design.

We added a flowchart of the design in figure 1 (method-design lines 218-219).

11. Why adolescents aged 12-23? You should explain this better, defining the term "adolescent", especially since there are social, mental and behavioural differences between the different stages of adolescence.

We added: *The age range is chosen to reflect Dutch clinical practice, where youth mental health services frequently provide specialized care for individuals up to the age of 23 to ensure continuity during the transition to adult life. To account for the wide age range, the analysis will differentiate between age categories based on three developmental substages early adolescence (12–14 years), middle adolescence (15–17 years), late adolescence/emerging adulthood (18–23 years).* See lines 247-252.

12. Is this protocol registered?
    The study protocol was not prospectively registered. As this is an inductive, qualitative study, it does not seek to test pre-defined hypotheses; therefore, the traditional rationale for registration—mitigating the risk of post-hoc hypothesis adjustment—is less applicable here than in deductive quantitative research. To ensure transparency and methodological rigor, we instead will employ continuous reflexivity and maintain a comprehensive record of all analytical choices made during the study.

My ethical concerns are clearly described, as are the limitations of this study.

**Reviewer #3:** This protocol delves into an aspect of DBT (especially in Adolescents),that could be elusive. It is a well constructed paper with exhaustive literature review and a solid framework for data collection and interviews.

Dear reviewer 3,

Thank you for your thoughtful questions. They helped us make our reasoning more explicit, thereby taking the quality of the study protocol to a higher level.

General questions

13. Have you considered individual therapist, and parental factors that could create bias and therfore confound results? (For example competence of the therapist?)

    Although we did not pre-specify these variables, therapist- and parent-related factors (such as age and gender) will be incorporated into the analysis. Regarding therapist competence, objective prior assessment is difficult. However, given that these clinicians work in a highly specialized setting and are extensively trained in Dialectical Behavior Therapy (DBT), we assume they possess the requisite skills to deliver the intervention effectively. To make our assumption more clear we added the sentence: *Regarding therapist competence, objective prior assessment is difficult; however, given that these clinicians work in a highly specialized setting and are extensively trained in DBT, we assume they possess the requisite skills to deliver the intervention effectively.* (See method-participants: lines 268-270). We also added the sentence: *Finally, to account for potential confounding effects, additional therapist- and parent-related factors (e.g., age and gender) will be incorporated into the analysis.* (See method-participants: lines 273-275.

14. Are we exploring the transferance-counter transference dynamic and it's effects? If so hasn't that been well documented.

We added the sentence: *Various dynamic factors have been identified as contributing to treatment success for adolescents with BPD features or are known in treatment success for adults with BPD features. These include the quality of the therapeutic alliance [8, 9], the emotional responses that adolescents and therapists hold toward each other also known as (counter) transference* (introduction: lines 161-164). We also made it more explicit in the topic list (see method-materials-interview guide line 364).

15. Since you've included ages from 12 to 23, which is a wide range of psychological development stages, do you plan on addressing the potential effect of age in the analysis and discussion?

We added: *The age range is chosen to reflect Dutch clinical practice, where youth mental health services frequently provide specialized care for individuals up to the age of 23 to ensure continuity during the transition to adult life. To account for the wide age range, the analysis will differentiate between age categories based on three developmental substages early adolescence (12–14 years), middle adolescence (15–17 years), late adolescence/emerging adulthood (18–23 years).* See Method-participants: lines 247-252

---

7. PLOS authors have the option to publish the peer review history of their article (what does this mean?). If published, this will include your full peer review and any attached files.

If you choose “no”, your identity will remain anonymous but your review may still be made public.

**Do you want your identity to be public for this peer review?** For information about this choice, including consent withdrawal, please see our Privacy Policy.

Reviewer #1: No

Reviewer #2: **Yes:** Patricia Alves

Reviewer #3: No

---
