# Supplementary material for: Unraveling dynamic factors in Dialectic Behavioural Treatment for Adolescents (DBT-A): A study protocol
Source: PLoS One. 2026 Jul 16;21(7):e0340173. doi: 10.1371/journal.pone.0340173 (PMC13375012; doi:10.1371/journal.pone.0340173)
Supplement: Attachment — Submitted filename: Response_to_Reviewers_auresp_2.pdf [file pone.0340173.s005.pdf]

Academic Editor Dr. Na

Plos One

Responsletter on manuscript PONE-D-25-63596R1

June 22, 2026

Dear Dr. Na,

Thank you for the opportunity to submit a revised version of our study protocol, 'Unraveling dynamic factors in Dialectic Behavioural Treatment for Adolescents (DBT-A): a study protocol (PONE-D-25-63596R1).

We greatly appreciate the time and constructive feedback.

We have carefully addressed your comments and incorporated the necessary revisions into the manuscript, as detailed in our point-by-point response below. For your convenience, we have uploaded both a clean copy and a tracked-changes version of the revised manuscript."

We hope that these revisions adequately address the concerns and that the manuscript is now suitable for publication in PLOS ONE.

Thank you again for your time, guidance, and consideration. We look forward to hearing from you.

Kind regards,

On behalf of all authors,

Anneke de Weerd

Point by point response

Although invitations were sent to the three reviewers who evaluated the original submission to review the revised manuscript, unfortunately none of them were able to accept the re-review request. Accordingly, as the handling editor, I personally examined the reviewers' major comments, the authors' responses, and the corresponding revisions made to the manuscript. Overall, the authors appear to have addressed the reviewers' concerns thoroughly and have substantially improved the manuscript. However, I believe the following two points would further strengthen the methodological clarity and transparency of the study.

#1. Regarding Reviewer #2, Comment 12

1. Recently, preregistration has increasingly been encouraged in qualitative research as well, as it can help clarify methodological and analytical decisions made prior to examining the actual data, and distinguish exploratory elements from a priori decision-making. Of course, given the nature of qualitative research, preregistration is not expected in the same manner as in quantitative hypothesis-testing studies, and it is not a mandatory requirement for publication in this journal.

   Nevertheless, if the authors ultimately decided not to preregister this study, I would ask them to state more explicitly in the manuscript the rationale for this decision, as well as what alternative measures (e.g., reflexivity procedures, audit trails, documentation of analytic decisions) will be used to ensure transparency and methodological rigor in place of preregistration.

We added after a paragraph mentioning the alternative measures.

> "*These steps serve as alternative measures to guarantee comprehensive transparency and methodological rigor in the absence of a prospectively registered study protocol. As an inductive, qualitative study, this research explores emergent themes rather than testing predefined hypotheses. Therefore, the primary rationale for preregistration, preventing post-hoc hypothesis adjustment, is less applicable in this context*.' Lines 424-428.

#2. Regarding Reviewer #3, Comment 13

2. The authors state that participating therapists work in a "highly specialized setting" and are "extensively trained" in DBT. However, as currently written, these descriptions may appear somewhat subjective, making it difficult for readers to objectively assess the therapists' level of expertise and training. Therefore, if possible, I encourage the authors to provide more concrete and objective criteria—such as DBT certification, formal training pathways, supervision structures, or minimum levels of clinical experience—which would further strengthen the methodological transparency and credibility of the study.

We added the sentence: *However, to ensure a high baseline standard of care, all participating therapists are required to hold a minimum of a bachelor's degree in a relevant clinical discipline and have completed formal DBT-A training. Furthermore, to maintain treatment fidelity, therapists actively participate in a DBT-A consultation team and receive both individual and team-based supervision from a certified DBT-A supervisor*. Lines 231-235.

We made more clear that we also want to collect the years of experience of an individual therapist: '*Finally, to account for potential confounding effects, additional therapist- and parent-related factors (e.g., age, gender, and the therapist's years of clinical experience as an individual therapist) will be incorporated into the analysis*.' Lines 240-243.
