# Supplementary material for: Unraveling dynamic factors in Dialectic Behavioural Treatment for Adolescents (DBT-A): A study protocol
Source: PLoS One. 2026 Jul 16;21(7):e0340173. doi: 10.1371/journal.pone.0340173 (PMC13375012; doi:10.1371/journal.pone.0340173)
Supplement: Peer review history

# Author comment: Author response to Decision Letter 0

**DOI:** 10.1371/journal.pone.0340173.r001

16 Dec 2025

Attachment Submitted filename: 3.1.3.4.2 Response letter_V1.pdf

# Peer review: Decision Letter 0

**DOI:** 10.1371/journal.pone.0340173.r002

**Authors:** Kyoung-Sae Na

25 Feb 2026

PONE-D-25-63596

Unraveling dynamic factors in Dialectic Behavioural Treatment for Adolescents (DBT-A): a study protocol

PLOS One

Dear Dr. de Weerd,

Thank you for submitting your manuscript to PLOS ONE. After careful consideration, we feel that it has merit but does not fully meet PLOS ONE’s publication criteria as it currently stands. Therefore, we invite you to submit a revised version of the manuscript that addresses the points raised during the review process.

==============================

The authors are to be commended for their efforts in preparing this manuscript. The three reviewers have now completed their evaluations. Overall, I found this protocol paper to be well structured and clearly written, and the authors demonstrate strong expertise in the field. The reviewers provided insightful methodological comments, particularly regarding aspects that would benefit from further clarification. In particular, the rationale for defining the participant age range as 12 to 23 years warrants more explicit explanation. An age span that bridges adolescence and adulthood is relatively uncommon in the DBT-A literature. If this age range reflects a context specific to the Dutch healthcare system (e.g., transitional youth services), it would be helpful to elaborate on this for readers from diverse international and cultural backgrounds, so that the applicability and rationale of this choice can be more clearly understood.

==============================

Please submit your revised manuscript by Apr 11 2026 11:59PM. If you will need more time than this to complete your revisions, please reply to this message or contact the journal office at plosone@plos.org. When you're ready to submit your revision, log on to https://www.editorialmanager.com/pone/ and select the ’submissions Needing Revision' folder to locate your manuscript file.

Please include the following items when submitting your revised manuscript:

- A letter that responds to each point raised by the academic editor and reviewer(s). You should upload this letter as a separate file labeled 'Response to Reviewers'.
- A marked-up copy of your manuscript that highlights changes made to the original version. You should upload this as a separate file labeled 'Revised Manuscript with Track Changes'.
- An unmarked version of your revised paper without tracked changes. You should upload this as a separate file labeled 'Manuscript'.

If you would like to make changes to your financial disclosure, please include your updated statement in your cover letter. Guidelines for resubmitting your figure files are available below the reviewer comments at the end of this letter.

If applicable, we recommend that you deposit your laboratory protocols in protocols.io to enhance the reproducibility of your results. Protocols.io assigns your protocol its own identifier (DOI) so that it can be cited independently in the future. For instructions see: https://journals.plos.org/plosone/s/submission-guidelines#loc-laboratory-protocols. Additionally, PLOS ONE offers an option for publishing peer-reviewed Lab Protocol articles, which describe protocols hosted on protocols.io. Read more information on sharing protocols at https://plos.org/protocols?utm_medium=editorial-email&utm_source=authorletters&utm_campaign=protocols.

We look forward to receiving your revised manuscript.

Kind regards,

Kyoung-Sae Na, M.D., Ph.D.

Academic Editor

PLOS One

Journal Requirements:

When submitting your revision, we need you to address these additional requirements.

1. Please ensure that your manuscript meets PLOS ONE’s style requirements, including those for file naming. The PLOS ONE style templates can be found at

https://journals.plos.org/plosone/s/file?id=wjVg/PLOSOne_formatting_sample_main_body.pdf and

https://journals.plos.org/plosone/s/file?id=ba62/PLOSOne_formatting_sample_title_authors_affiliations.pdf.

2. Please be informed that funding information should not appear in the Acknowledgments section or other areas of your manuscript. We will only publish funding information present in the Funding Statement section of the online submission form. Please remove any funding-related text from the manuscript.

3. If the reviewer comments include a recommendation to cite specific previously published works, please review and evaluate these publications to determine whether they are relevant and should be cited. There is no requirement to cite these works unless the editor has indicated otherwise.

[Note: HTML markup is below. Please do not edit.]

Reviewers' comments:

Reviewer’s Responses to Questions

**Comments to the Author**

1. Does the manuscript provide a valid rationale for the proposed study, with clearly identified and justified research questions?

The research question outlined is expected to address a valid academic problem or topic and contribute to the base of knowledge in the field.

Reviewer #1: Yes

Reviewer #2: Yes

Reviewer #3: Yes

**********

2. Is the protocol technically sound and planned in a manner that will lead to a meaningful outcome and allow testing the stated hypotheses?

The manuscript should describe the methods in sufficient detail to prevent undisclosed flexibility in the experimental procedure or analysis pipeline, including sufficient outcome-neutral conditions (e.g. necessary controls, absence of floor or ceiling effects) to test the proposed hypotheses and a statistical power analysis where applicable. As there may be aspects of the methodology and analysis which can only be refined once the work is undertaken, authors should outline potential assumptions and explicitly describe what aspects of the proposed analyses, if any, are exploratory.

Reviewer #1: Yes

Reviewer #2: Yes

Reviewer #3: Yes

**********

3. Is the methodology feasible and described in sufficient detail to allow the work to be replicable?

Descriptions of methods and materials in the protocol should be reported in sufficient detail for another researcher to reproduce all experiments and analyses. The protocol should describe the appropriate controls, sample size calculations, and replication needed to ensure that the data are robust and reproducible.

Reviewer #1: Yes

Reviewer #2: Yes

Reviewer #3: Yes

**********

4. Have the authors described where all data underlying the findings will be made available when the study is complete?

The PLOS Data policy requires authors to make all data underlying the findings described in their manuscript fully available without restriction, with rare exception, at the time of publication. The data should be provided as part of the manuscript or its supporting information, or deposited to a public repository. For example, in addition to summary statistics, the data points behind means, medians and variance measures should be available. If there are restrictions on publicly sharing data—e.g. participant privacy or use of data from a third party—those must be specified.

Reviewer #1: No

Reviewer #2: Yes

Reviewer #3: Yes

**********

5. Is the manuscript presented in an intelligible fashion and written in standard English?

PLOS ONE does not copyedit accepted manuscripts, so the language in submitted articles must be clear, correct, and unambiguous. Any typographical or grammatical errors should be corrected at revision, so please note any specific errors here.

Reviewer #1: Yes

Reviewer #2: Yes

Reviewer #3: Yes

**********

6. Review Comments to the Author

Please use the space provided to explain your answers to the questions above and, if applicable, provide comments about issues authors must address before this protocol can be accepted for publication. You may also include additional comments for the author, including concerns about research or publication ethics.

You may also provide optional suggestions and comments to authors that they might find helpful in planning their study.

(Please upload your review as an attachment if it exceeds 20,000 characters)

Reviewer #1: Dear Authors,

Thank you for the opportunity to review your study protocol. I would like to begin by acknowledging the significant value of this work.

Below are my formal comments and the issues that should be addressed before the protocol can be considered for publication.

1. Rationale and Research Questions

The manuscript provides a valid and well-justified rationale. By moving beyond aggregate quantitative data to explore individual "trajectories," you address a critical gap in understanding why some adolescents do not respond to DBT-A. The research questions are clearly stated and align with the proposed qualitative longitudinal design.

2. Methodology and Replicability

The protocol is technically sound and the use of Reflexive Thematic Analysis (RTA) combined with Framework Analysis is appropriate. However, to ensure full replicability, the following points must be addressed:

Interview Guides: While you identify themes like "alliance" and "motivation," the specific semi-structured interview prompts are not included. Please provide the interview topic guides as supporting information.

Definition of a "Case": Given the high-risk nature of this population, dropout is likely. You must explicitly define what constitutes a "complete case" for your analysis. If a parent or therapist leaves the study but the adolescent remains, will that trajectory still be included? Clarifying the "minimum data set" is essential to prevent undisclosed flexibility in the analysis pipeline.

3. Feasibility and Potential Biases

Recruitment Bias: Because therapists recruit their own patients, there is a significant risk of "cherry-picking" stable or "likable" cases. I suggest the authors describe a more randomized recruitment approach (e.g., inviting the next three eligible patients) to ensure the data includes the "difficult" dynamics the study aims to unravel.

Parental Dyads: You mention a preference for interviewing parents together. This "dyadic" approach can suppress conflicting views or domestic tensions. Please justify this choice or mention a protocol for offering individual "break-out" sessions if tension is observed.

4. Data Availability and Ethics

PLOS Data Policy: You have correctly identified that the data is sensitive and will require restricted access. However, for publication, you must provide contact information for an independent Data Access Committee or Ethics Committee. Relying solely on "available upon request from the authors" does not meet current transparency standards.

Sentence Completion: Please review page 13; the sentence ending in "...and to" appears to be cut off.

5. Language and Presentation

The manuscript is written in high-quality standard English. Your disclosure regarding the use of AI for translation and linguistic refinement is appreciated and aligns with ethical transparency, provided you maintain full accountability for the technical content.

Optional Suggestions for the Study Plan

Member Checking: To mitigate attrition over the 9-month period, consider offering participants a brief, clinically appropriate summary of the general themes found in the study as an incentive for their continued contribution.

Power Dynamics: In the triadic model, the researcher must be careful not to be perceived as "part of the clinical team." Clearly emphasizing the independence of the research team during the consent process will be vital for obtaining honest critiques of the therapy.

Reviewer #2: A very interesting study with relevant objectives. Congratulations!

Methodology:

It would be important to better describe the type of study. From what I understand, it is a mixed, prospective study with interviews, questionnaires, and scales.

Why not put it in a flowchart? To better understand the study design.

Why adolescents aged 12-23? You should explain this better, defining the term “adolescent”, especially since there are social, mental and behavioural differences between the different stages of adolescence.

Is this protocol registered?

My ethical concerns are clearly described, as are the limitations of this study.

Reviewer #3: This protocol delves into an aspect of DBT (especially in Adolescents),that could be elusive. It is a well constructed paper with exhaustive literature review and a solid framework for data collection and interviews.

General questions

Have you considered individual therapist, and parental factors that could create bias and therfore confound results? (For example competence of the therapist?)

Are we exploring the transferance-counter transference dynamic and it’s effects? If so hasn't that been well documented.

Since you've included ages from 12 to 23, which is a wide range of psychological development stages, do you plan on addressing the potential effect of age in the analysis and discussion?

**********

7. PLOS authors have the option to publish the peer review history of their article (what does this mean?). If published, this will include your full peer review and any attached files.

If you choose “no”, your identity will remain anonymous but your review may still be made public.

**Do you want your identity to be public for this peer review?**  For information about this choice, including consent withdrawal, please see our Privacy Policy.

Reviewer #1: No

Reviewer #2: **Yes:** Patricia Alves

Reviewer #3: No

**********

[NOTE: If reviewer comments were submitted as an attachment file, they will be attached to this email and accessible via the submission site. Please log into your account, locate the manuscript record, and check for the action link "View Attachments". If this link does not appear, there are no attachment files.]

To ensure your figures meet our technical requirements, please review our figure guidelines: https://journals.plos.org/plosone/s/figures

You may also use PLOS’s free figure tool, NAAS, to help you prepare publication quality figures: https://journals.plos.org/plosone/s/figures#loc-tools-for-figure-preparation.

NAAS will assess whether your figures meet our technical requirements by comparing each figure against our figure specifications.

# Author comment: Author response to Decision Letter 1

**DOI:** 10.1371/journal.pone.0340173.r003

31 Mar 2026

Academic Editor Dr. Na

Plos One

Responsletter on manuscript [PONE-D-25-63596]

March 29, 2026

Dear Dr. Na,

Thank you for giving us the opportunity to submit a revised version of our study protocol titled "Unraveling dynamic factors in Dialectic Behavioural Treatment for Adolescents (DBT-A): a study protocol" (PONE-D-25-63596). We sincerely appreciate the time and effort you and the reviewers have dedicated to reviewing our work. The constructive feedback has been highly valuable and has helped us to significantly improve the quality and clarity of our protocol.

We have carefully considered all the comments provided by the reviewers and have made the necessary improvements and additions to the manuscript in the point-by-point responses below.

We have also uploaded both a clean copy of the revised manuscript and a version with 'tracked changes' so that the modifications are easy to identify. Additionally, we have included the interview guide as a supplementary file. This document outlines the specific questions that will be asked to the participants, systematically organized by topic.

We hope that these revisions adequately address the reviewers' concerns and that the manuscript is now suitable for publication in Plos One.

Thank you again for your time, guidance, and consideration. We look forward to hearing from you.

Kind regards,

On behalf of all authors,

Anneke de Weerd

Reviewer #1: Dear Authors, Thank you for the opportunity to review your study protocol. I would like to begin by acknowledging the significant value of this work. Below are my formal comments and the issues that should be addressed before the protocol can be considered for publication.

Dear Reviewer 1,

Thank you for your thorough review and your highly insightful comments. Your feedback has been invaluable in adding necessary depth to this section of our protocol. See below our response/adaptions to the manuscript.

1. Rationale and Research Questions The manuscript provides a valid and well-justified rationale. By moving beyond aggregate quantitative data to explore individual "trajectories," you address a critical gap in understanding why some adolescents do not respond to DBT-A. The research questions are clearly stated and align with the proposed qualitative longitudinal design.

Thank you. 2. Methodology and Replicability The protocol is technically sound and the use of Reflexive Thematic Analysis (RTA) combined with Framework Analysis is appropriate. However, to ensure full replicability, the following points must be addressed:

1.

Interview Guides: While you identify themes like "alliance" and "motivation," the specific semi-structured interview prompts are not included. Please provide the interview topic guides as supporting information.

We added an additional file with the interview prompts included for all participants groups (see supporting information 2). We added a reference in the study protocol to the document in Method-materials at line 356.

2.

Definition of a "Case": Given the high-risk nature of this population, dropout is likely. You must explicitly define what constitutes a "complete case" for your analysis. If a parent or therapist leaves the study but the adolescent remains, will that trajectory still be included? Clarifying the "minimum data set" is essential to prevent

undisclosed flexibility in the analysis pipeline.

We added the sentence: A complete case is defined by a minimum of three measurements moments per participant group (e.g. adolescent, parent and individual therapist). Although incomplete cases/trajectories are excluded from the longitudinal analysis, their individual data points will be included in thematic sub-analyses to maximize data utility. (see Participants lines 284-287).

3. Feasibility and Potential Biases

3.

Recruitment Bias: Because therapists recruit their own patients, there is a significant risk of "cherry-picking" stable or "likable" cases. I suggest the authors describe a more randomized recruitment approach (e.g., inviting the next three eligible patients) to ensure the data includes the "difficult" dynamics the study aims to unravel.

Based on the eligibility criteria, these contacts will screen all adolescents admitted to DBT-A treatment between 2025 and 2026 and all will be invited to participate in order to minimize selection bias. We also added: The contact person at each participating institution will maintain a screening record of all potential participants, documenting their eligibility status and the rationale for inclusion or exclusion (lines 299-302)

4.

Parental Dyads: You mention a preference for interviewing parents together. This "dyadic" approach can suppress conflicting views or domestic tensions. Please justify this choice or mention a protocol for offering individual "break-out" sessions if tension is observed.

We rewrote and added the advise to: Parents are offered the choice of joint or individual participation. Although both parents will be interviewed together in a dyadic format where possible, individual interviews will be arranged if a joint session is not preferred. If participation from both parents is not feasible, the involvement of a single parent is sufficient. (see method-participants lines 261-265).

4. Data Availability and Ethics

5.

PLOS Data Policy: You have correctly identified that the data is sensitive and will require restricted access. However, for publication, you must provide contact information for an independent Data Access Committee or Ethics Committee. Relying solely on "available upon request from the authors" does not meet current

transparency standards.

We added the sentence: The pseudonymized datasets generated during the current study will be available from the LUMC Curium data officer upon reasonable request, subject to consultation with the authors. (meta data-see lines 27-29).

6.

Sentence Completion: Please review page 13; the sentence ending in "...and to" appears to be cut off.

We have re-read the manuscript but cannot find the sentence completion issue the reviewer is pointing at.

5. Language and Presentation The manuscript is written in high-quality standard English. Your disclosure regarding the use of AI for translation and linguistic refinement is appreciated and aligns with ethical transparency, provided you maintain full accountability for the technical content.

Thank you, we indeed take full accountability for the technical content, and we have added this to the statement (metadata- see line 35).

7.

Optional Suggestions for the Study Plan Member Checking: To mitigate attrition over the 9-month period, consider offering participants a brief, clinically appropriate summary of the general themes found in the study as an incentive for their continued contribution.

Thank you for this suggestion. To further explain the procedure, the expert by experience and the project team, representing all four participating institutions, will validate the themes emerging from the analysis. Participants will be offered the opportunity to review their transcripts for accuracy and provide clarifications upon completion of the data collection. Furthermore, the overall study results will be shared with participants. Given the longitudinal design and the need to avoid biasing participants during the nine-month study period, this information will be provided only after the study has concluded. This has been noted in lines the protocol, under the 'Procedure' section lines 326-328.

8.

Power Dynamics: In the triadic model, the researcher must be careful not to be perceived as "part of the clinical team." Clearly emphasizing the independence of the research team during the consent process will be vital for obtaining honest critiques of the therapy.

We added the following information to emphasize the independence: The researcher will explicitly declare her independence from the clinical team to mitigate power imbalances. Additionally, participants will be assured of intra-triadic confidentiality, clarifying that no

information provided by one participant will be disclosed to the others during the research process. (see lines 310-313 in procedure).

Reviewer #2: A very interesting study with relevant objectives. Congratulations! Dear Reviewer 2,

Thank you for the points raised below. Your detailed feedback offered a valuable perspective, allowing us to strengthen and elaborate on several aspects of our methodology.

Methodology:

9.

It would be important to better describe the type of study. From what I understand, it is a mixed, prospective study with interviews, questionnaires, and scales.

We added: This study adopts a prospective, longitudinal multiple-case design with an embedded mixed methods approach. A qualitatively-driven interpretation will be employed, placing primary emphasis on the qualitative findings, while quantitative data serve a supportive, complementary role. Lines 201-204

10.

Why not put it in a flowchart? To better understand the study design.

We added a flowchart of the design in figure 1 (method-design lines 218-219).

11.

Why adolescents aged 12-23? You should explain this better, defining the term “adolescent”, especially since there are social, mental and behavioural differences between the different stages of adolescence.

We added: The age range is chosen to reflect Dutch clinical practice, where youth mental health services frequently provide specialized care for individuals up to the age of 23 to ensure continuity during the transition to adult life. To account for the wide age range, the analysis will differentiate between age categories based on three developmental substages early adolescence (12–14 years), middle adolescence (15–17 years), late adolescence/emerging adulthood (18–23 years). See lines 247-252.

12.

Is this protocol registered? The study protocol was not prospectively registered. As this is an inductive, qualitative study, it does not seek to test pre-defined hypotheses; therefore, the traditional rationale for registration—mitigating the risk of post-hoc hypothesis adjustment—is less applicable here than in deductive quantitative research. To ensure transparency and methodological rigor, we instead will employ continuous reflexivity and maintain a comprehensive record of all analytical choices made during the study.

My ethical concerns are clearly described, as are the limitations of this study.

Reviewer #3: This protocol delves into an aspect of DBT (especially in Adolescents),that could be elusive. It is a well constructed paper with exhaustive literature review and a solid framework for data collection and interviews.

Dear reviewer 3,

Thank you for your thoughtful questions. They helped us make our reasoning more explicit, thereby taking the quality of the study protocol to a higher level.

General questions

13.

Have you considered individual therapist, and parental factors that could create bias and therfore confound results? (For example competence of the therapist?)

Although we did not pre-specify these variables, therapist- and parent-related factors (such as age and gender) will be incorporated into the analysis. Regarding therapist competence, objective prior assessment is difficult. However, given that these clinicians work in a highly specialized setting and are extensively trained in Dialectical Behavior Therapy (DBT), we assume they possess the requisite skills to deliver the intervention effectively. To make our assumption more clear we added the sentence: Regarding therapist competence, objective prior assessment is difficult; however, given that these clinicians work in a highly specialized setting and are extensively trained in DBT, we assume they possess the requisite skills to deliver the intervention effectively. (See method-participants: lines 268-270). We also added the sentence: Finally, to account for potential confounding effects, additional therapist- and parent-related factors (e.g., age and gender) will be incorporated into the analysis. (See method-participants: lines 273-275.

14.

Are we exploring the transferance-counter transference dynamic and it’s effects? If so hasn't that been well documented.

We added the sentence: Various dynamic factors have been identified as contributing to treatment success for adolescents with BPD features or are known in treatment success for adults with BPD features. These include the quality of the therapeutic alliance [8, 9], the emotional responses that adolescents and therapists hold toward each other also known as (counter) transference (introduction: lines 161-164). We also made it more explicit in the topic list (see method-materials-interview guide line 364).

15.

Since you've included ages from 12 to 23, which is a wide range of psychological development stages, do you plan on addressing the potential effect of age in the analysis and discussion?

We added: The age range is chosen to reflect Dutch clinical practice, where youth mental health services frequently provide specialized care for individuals up to the age of 23 to ensure continuity during the transition to adult life. To account for the wide age range, the analysis will differentiate between age categories based on three developmental substages early adolescence (12–14 years), middle adolescence (15–17 years), late adolescence/emerging adulthood (18–23 years). See Method-participants: lines 247-252

Attachment Submitted filename: Response to Reviewers.pdf

# Editor report: Decision Letter 1

**DOI:** 10.1371/journal.pone.0340173.r004

**Authors:** Kyoung-Sae Na, Kyoung-Sae Na

25 May 2026

PONE-D-25-63596R1

Unraveling dynamic factors in Dialectic Behavioural Treatment for Adolescents (DBT-A): a study protocol

PLOS One

Dear Dr. de Weerd,

Thank you for submitting your manuscript to PLOS ONE. After careful consideration, we feel that it has merit but does not fully meet PLOS ONE’s publication criteria as it currently stands. Therefore, we invite you to submit a revised version of the manuscript that addresses the points raised during the review process.

==============================

Although invitations were sent to the three reviewers who evaluated the original submission to review the revised manuscript, unfortunately none of them were able to accept the re-review request. Accordingly, as the handling editor, I personally examined the reviewers’ major comments, the authors’ responses, and the corresponding revisions made to the manuscript. Overall, the authors appear to have addressed the reviewers’ concerns thoroughly and have substantially improved the manuscript. However, I believe the following two points would further strengthen the methodological clarity and transparency of the study.

#1. Regarding Reviewer #2, Comment 12

Recently, preregistration has increasingly been encouraged in qualitative research as well, as it can help clarify methodological and analytical decisions made prior to examining the actual data, and distinguish exploratory elements from a priori decision-making. Of course, given the nature of qualitative research, preregistration is not expected in the same manner as in quantitative hypothesis-testing studies, and it is not a mandatory requirement for publication in this journal.

Nevertheless, if the authors ultimately decided not to preregister this study, I would ask them to state more explicitly in the manuscript the rationale for this decision, as well as what alternative measures (e.g., reflexivity procedures, audit trails, documentation of analytic decisions) will be used to ensure transparency and methodological rigor in place of preregistration.

<h4>#2. Regarding Reviewer #3, Comment 13</h4>

The authors state that participating therapists work in a “highly specialized setting” and are “extensively trained” in DBT. However, as currently written, these descriptions may appear somewhat subjective, making it difficult for readers to objectively assess the therapists’ level of expertise and training. Therefore, if possible, I encourage the authors to provide more concrete and objective criteria—such as DBT certification, formal training pathways, supervision structures, or minimum levels of clinical experience—which would further strengthen the methodological transparency and credibility of the study.

==============================

Please submit your revised manuscript by Jul 09 2026 11:59PM. If you will need more time than this to complete your revisions, please reply to this message or contact the journal office at plosone@plos.org. When you're ready to submit your revision, log on to https://www.editorialmanager.com/pone/ and select the ’submissions Needing Revision' folder to locate your manuscript file.

Please include the following items when submitting your revised manuscript:

- A letter that responds to each point raised by the academic editor and reviewer(s). You should upload this letter as a separate file labeled 'Response to Reviewers'.
- A marked-up copy of your manuscript that highlights changes made to the original version. You should upload this as a separate file labeled 'Revised Manuscript with Track Changes'.
- An unmarked version of your revised paper without tracked changes. You should upload this as a separate file labeled 'Manuscript'.

If you would like to make changes to your financial disclosure, please include your updated statement in your cover letter. Guidelines for resubmitting your figure files are available below the reviewer comments at the end of this letter.

If applicable, we recommend that you deposit your laboratory protocols in protocols.io to enhance the reproducibility of your results. Protocols.io assigns your protocol its own identifier (DOI) so that it can be cited independently in the future. For instructions see: https://journals.plos.org/plosone/s/submission-guidelines#loc-laboratory-protocols. Additionally, PLOS ONE offers an option for publishing peer-reviewed Lab Protocol articles, which describe protocols hosted on protocols.io. Read more information on sharing protocols at https://plos.org/protocols?utm_medium=editorial-email&utm_source=authorletters&utm_campaign=protocols.

As the corresponding author, your ORCID iD is verified in the submission system and will appear in the published article. PLOS supports the use of ORCID, and we encourage all coauthors to register for an ORCID iD and use it as well. Please encourage your coauthors to verify their ORCID iD within the submission system before final acceptance, as unverified ORCID iDs will not appear in the published article. *Only*  the individual author can complete the verification step; PLOS staff *cannot*  verify ORCID iDs on behalf of authors.

We look forward to receiving your revised manuscript.

Kind regards,

Kyoung-Sae Na, M.D., Ph.D.

Academic Editor

PLOS One

Journal Requirements:

If the reviewer comments include a recommendation to cite specific previously published works, please review and evaluate these publications to determine whether they are relevant and should be cited. There is no requirement to cite these works unless the editor has indicated otherwise.

Please review your reference list to ensure that it is complete and correct. If you have cited papers that have been retracted, please include the rationale for doing so in the manuscript text, or remove these references and replace them with relevant current references. Any changes to the reference list should be mentioned in the rebuttal letter that accompanies your revised manuscript. If you need to cite a retracted article, indicate the article’s retracted status in the References list and also include a citation and full reference for the retraction notice.

[Note: HTML markup is below. Please do not edit.]

[NOTE: If reviewer comments were submitted as an attachment file, they will be attached to this email and accessible via the submission site. Please log into your account, locate the manuscript record, and check for the action link "View Attachments". If this link does not appear, there are no attachment files.]

To ensure your figures meet our technical requirements, please review our figure guidelines: https://journals.plos.org/plosone/s/figures

You may also use PLOS’s free figure tool, NAAS, to help you prepare publication quality figures: https://journals.plos.org/plosone/s/figures#loc-tools-for-figure-preparation.

NAAS will assess whether your figures meet our technical requirements by comparing each figure against our figure specifications.

# Author comment: Author response to Decision Letter 2

**DOI:** 10.1371/journal.pone.0340173.r005

25 Jun 2026

Academic Editor Dr. Na

Plos One

Responsletter on manuscript PONE-D-25-63596R1

June 22, 2026

Dear Dr. Na,

Thank you for the opportunity to submit a revised version of our study protocol, 'Unraveling dynamic factors in Dialectic Behavioural Treatment for Adolescents (DBT-A): a study protocol (PONE-D-25-63596R1).

We greatly appreciate the time and constructive feedback.

We have carefully addressed your comments and incorporated the necessary revisions into the manuscript, as detailed in our point-by-point response below. For your convenience, we have uploaded both a clean copy and a tracked-changes version of the revised manuscript."

We hope that these revisions adequately address the concerns and that the manuscript is now suitable for publication in PLOS ONE.

Thank you again for your time, guidance, and consideration. We look forward to hearing from you.

Kind regards,

On behalf of all authors,

Anneke de Weerd

Point by point response

Although invitations were sent to the three reviewers who evaluated the original submission to review the revised manuscript, unfortunately none of them were able to accept the re-review request. Accordingly, as the handling editor, I personally examined the reviewers’ major comments, the authors’ responses, and the corresponding revisions made to the manuscript. Overall, the authors appear to have addressed the reviewers’ concerns thoroughly and have substantially improved the manuscript. However, I believe the following two points would further strengthen the methodological clarity and transparency of the study.

#1. Regarding Reviewer #2, Comment 12

1. Recently, preregistration has increasingly been encouraged in qualitative research as well, as it can help clarify methodological and analytical decisions made prior to examining the actual data, and distinguish exploratory elements from a priori decision-making. Of course, given the nature of qualitative research, preregistration is not expected in the same manner as in quantitative hypothesis-testing studies, and it is not a mandatory requirement for publication in this journal.

Nevertheless, if the authors ultimately decided not to preregister this study, I would ask them to state more explicitly in the manuscript the rationale for this decision, as well as what alternative measures (e.g., reflexivity procedures, audit trails, documentation of analytic decisions) will be used to ensure transparency and methodological rigor in place of preregistration.

We added after a paragraph mentioning the alternative measures.

“These steps serve as alternative measures to guarantee comprehensive transparency and methodological rigor in the absence of a prospectively registered study protocol. As an inductive, qualitative study, this research explores emergent themes rather than testing predefined hypotheses. Therefore, the primary rationale for preregistration, preventing post-hoc hypothesis adjustment, is less applicable in this context.’ Lines 424-428.

#2. Regarding Reviewer #3, Comment 13

2. The authors state that participating therapists work in a “highly specialized setting” and are “extensively trained” in DBT. However, as currently written, these descriptions may appear somewhat subjective, making it difficult for readers to objectively assess the therapists’ level of expertise and training. Therefore, if possible, I encourage the authors to provide more concrete and objective criteria—such as DBT certification, formal training pathways, supervision structures, or minimum levels of clinical experience—which would further strengthen the methodological transparency and credibility of the study.

We added the sentence: However, to ensure a high baseline standard of care, all participating therapists are required to hold a minimum of a bachelor’s degree in a relevant clinical discipline and have completed formal DBT-A training. Furthermore, to maintain treatment fidelity, therapists actively participate in a DBT-A consultation team and receive both individual and team-based supervision from a certified DBT-A supervisor. Lines 231-235.

We made more clear that we also want to collect the years of experience of an individual therapist: ‘Finally, to account for potential confounding effects, additional therapist- and parent-related factors (e.g., age, gender, and the therapist’s years of clinical experience as an individual therapist) will be incorporated into the analysis.’ Lines 240-243.

Attachment Submitted filename: Response_to_Reviewers_auresp_2.pdf

# Editor report: Decision Letter 2

**DOI:** 10.1371/journal.pone.0340173.r006

**Authors:** Kyoung-Sae Na, Kyoung-Sae Na, Kyoung-Sae Na

29 Jun 2026

Unraveling dynamic factors in Dialectic Behavioural Treatment for Adolescents (DBT-A): a study protocol

PONE-D-25-63596R2

Dear Dr. de Weerd,

We’re pleased to inform you that your manuscript has been judged scientifically suitable for publication and will be formally accepted for publication once it meets all outstanding technical requirements.

Within one week, you’ll receive an e-mail detailing the required amendments. When these have been addressed, you’ll receive a formal acceptance letter and your manuscript will be scheduled for publication.

An invoice will be generated when your article is formally accepted. Please note, if your institution has a publishing partnership with PLOS and your article meets the relevant criteria, all or part of your publication costs will be covered. Please make sure your user information is up-to-date by logging into Editorial Manager at Editorial Manager® and clicking the ‘Update My Information' link at the top of the page. For questions related to billing, please contact billing support.

If your institution or institutions have a press office, please notify them about your upcoming paper to help maximize its impact. If they’ll be preparing press materials, please inform our press team as soon as possible -- no later than 48 hours after receiving the formal acceptance. Your manuscript will remain under strict press embargo until 2 pm Eastern Time on the date of publication. For more information, please contact onepress@plos.org.

Kind regards,

Kyoung-Sae Na, M.D., Ph.D.

Academic Editor

PLOS One

Additional Editor Comments (optional):

Reviewers' comments:

# Editor report: Acceptance letter

**DOI:** 10.1371/journal.pone.0340173.r007

**Authors:** Kyoung-Sae Na, Kyoung-Sae Na, Kyoung-Sae Na

PONE-D-25-63596R2

PLOS One

Dear Dr. de Weerd,

I'm pleased to inform you that your manuscript has been deemed suitable for publication in PLOS One. Congratulations! Your manuscript is now being handed over to our production team.

At this stage, our production department will prepare your paper for publication. This includes ensuring the following:

* All references, tables, and figures are properly cited

* All relevant supporting information is included in the manuscript submission,

* There are no issues that prevent the paper from being properly typeset

You will receive further instructions from the production team, including instructions on how to review your proof when it is ready. Please keep in mind that we are working through a large volume of accepted articles, so please give us a few days to review your paper and let you know the next and final steps.

Lastly, if your institution or institutions have a press office, please let them know about your upcoming paper now to help maximize its impact. If they'll be preparing press materials, please inform our press team within the next 48 hours. Your manuscript will remain under strict press embargo until 2 pm Eastern Time on the date of publication. For more information, please contact onepress@plos.org.

You will receive an invoice from PLOS for your publication fee after your manuscript has reached the completed accept phase. If you receive an email requesting payment before acceptance or for any other service, this may be a phishing scheme. Learn how to identify phishing emails and protect your accounts at https://explore.plos.org/phishing.

If we can help with anything else, please email us at customercare@plos.org.

Thank you for submitting your work to PLOS ONE and supporting open access.

Kind regards,

PLOS ONE Editorial Office Staff

on behalf of

Dr. Kyoung-Sae Na

Academic Editor

PLOS One
